# Attenuation by Time-Restricted Feeding of High-Fat and High-Fructose Diet-Induced NASH in Mice Is Related to Per2 and Ferroptosis

**DOI:** 10.1155/2022/8063897

**Published:** 2022-10-15

**Authors:** Yan-yun Shu, Wen-kang Gao, Hui-kuan Chu, Ling Yang, Xiao-li Pan, Jin Ye

**Affiliations:** Division of Gastroenterology, Union Hospital, Tongji Medical College, Huazhong University of Science and Technology, Wuhan, China 430022

## Abstract

Nonalcoholic steatohepatitis (NASH) is a chronic and progressive disease whose treatment strategies are limited. Although time-restricted feeding (TRF) is beneficial for metabolic diseases without influencing caloric intake, the underlying mechanisms of TRF action in NASH and its efficacy have not yet been demonstrated. We herein showed that TRF effectively alleviated NASH, producing a reduction in liver enzymes and improvements in liver pathology. Regarding the mechanisms by which TRF mitigates NASH, we ascertained that TRF inhibited ferroptosis and the expression of the circadian gene Per2. By adopting a hepatocyte-specific Per2-knockout (Per2^△hep^) mice model, we clarified the critical role of Per2 in exacerbating NASH. According to the results of our RNA-Seq analysis, the knockout of Per2 ameliorated NASH by inhibiting the onset of ferroptosis; this was manifested by diminished lipid peroxidation levels, decreased mRNA and protein levels for ferroptosis-related genes, and alleviated morphologic changes in mitochondria. Furthermore, using a ferroptosis inhibitor, we showed that ferroptosis significantly aggravated NASH and noted that this was likely achieved by regulation of the expression of peroxisome proliferator activated receptor (PPAR)*α*. Finally, we discerned that TRF and hepatocyte-specific knockout of Per2 promoted the expression of PPAR*α*. Our results revealed a potential for TRF to effectively alleviate high-fat and high-fructose diet-induced NASH via the inhibition of Per2 and depicted the participation of Per2 in the progression of NASH by promoting ferroptosis, which was ultimately related to the expression of PPAR*α*.

## 1. Introduction

Nonalcoholic steatohepatitis (NASH), a progressive form of nonalcoholic fatty liver disease (NAFLD), is a chronic and progressive disease characterized by accumulated liver fat, hepatocyte injury, and inflammatory infiltration [1, 2]. The advent of fast-paced lifestyles and high-calorie diets has enhanced the incidence rate of metabolic diseases [3, 4], contributing to an increased incidence rate of NAFLD. Epidemiologic studies have indicated that the global incidence rate of NAFLD is nearly 25%, and this contributes to the increasing prevalence of NASH [5]. NASH eventually increases the risk of developing end-stage liver disease and places a great burden on society as a whole [3, 4]. There is therefore an acute need for the elucidation of definitive pathogenesis and effective treatment concerning NASH.

Lifestyle modifications—for example, reducing calorie intake or increasing exercise—constitute the core of NASH therapy; although time-restricted feeding (TRF) is considered to exert some effects on NASH [6, 7], their efficacy and underlying pathophysiologic mechanisms of action remain unclear. TRF refers to regular intermittent calorie control, implying that food intake remains restricted to fixed hours without deliberately reducing calorie intake, while during other hours of the day, food is not made available. Clinical research has shown that intermittent fasting (also described as TRF) is an effective strategy for weight loss and metabolic-index improvement [8, 9]. While exploring the mechanism of TRF action in metabolic disease, some animal studies showed that TRF protected against weight gain and improved glucose tolerance and lipid metabolism [6, 10]. Furthermore, TRF also protected mice from liver steatosis and damage, without influencing the total calorie intake, when they were fed a high-fat diet [6]. These researchers subsequently ascertained that TRF restored the expression of the circadian genes [6], suggesting the possibility that the effectiveness of TRF was achieved by regulating the expression of such genes.

A pivotal role of the circadian clock in the pathogenesis and progression of NASH has been investigated in recent years [11, 12]. Per2, a member of the circadian gene family, is important in modulating the circadian rhythm and the expression of other circadian genes [13, 14]. Previous studies revealed that mice lacking the Per2 gene or the functional Per2 protein exhibited reductions in body weight, total plasma triacylglycerol, and plasma glucose levels [15, 16]—indicating an important role for Per2 in the metabolism of glucose and fat. Considering that TRF influences the expression of circadian genes [6], we hypothesized that the beneficial effects of TRF were achieved by regulating the Per2 gene. However, whether Per2 participates in NASH and its underlying mechanisms are not clear.

Ferroptosis, a newly uncovered form of cell death, is a type of regulated cell death characterized by iron-dependent accumulation of lipid peroxidation (LPO) [17], which is also related to the pathogenesis and progression of NASH. In addition to its effects on cancer and stroke [18, 19], the ferroptosis is reported to be crucial in triggering inflammation [20, 21] as well as promoting the formation of lipid droplets in NASH [20]. In 2019, researchers demonstrated the promotion of ferroptosis after autophagic degradation of certain circadian genes [22], indicating that ferroptosis might be regulated by such genes.

The family of peroxisome proliferator-activated receptors (PPARs) is significant in regulating body fat and energy metabolism and is reported to occupy a critical position in the progression of NASH [23, 24]. Regarding the subtypes of PPARs, previous investigators reported that PPAR*α* and PPAR*γ* were directly or indirectly regulated by the Per2 [15, 25] and that the activity of PPAR*α* might influence susceptibility to ferroptosis [26]. The aforementioned observations suggested a possible relationship between Per2, ferroptosis, and PPARs in the pathophysiology of NASH.

We therefore herein intended to investigate the efficacy and mechanisms by which TRF alleviated NASH, the relationship between Per2 and ferroptosis, and the mechanism by which Per2 and ferroptosis participated in NASH.

## 2. Materials and Methods

### 2.1. Animals and Diets

This animal experiment was approved by the Ethics Committee for Animal Experimentation (IACUC number 2818), and all animals received humane care in accordance with the National Institutes of Health Guide for the Care and Use of Laboratory Animals (NIH Publications No. 8023, revised, 1978). Male white-type C57BL/6J mice were purchased from Beijing Vital River Laboratory Animal Technology Co., Ltd., and the Per2 double-floxed mice were generated at GemPharmatech Co., Ltd. on a C57BL6J background. An AVV8-TGB-iCre vector (Wuhan Qijing Biotechnology Co., Ltd., China) was injected intravenously (iv) into the tails of Per2 double-floxed mice to generate hepatocyte-specific Per2-knockout (Per2^△hep^) mice, while the AVV8-TGB- MCS-wpre vector (Wuhan Qijing Biotechnology Co., Ltd., China) was also tail injected iv into Per2 double-floxed mice to generate the controls (Per2^fl/fl^). After acclimating to their experimental environment with a normal chow diet for one week, the aforementioned mice (age 8 weeks) were raised for 16 weeks in a 12 h light/12 h dark cycle on a high-fat and high-fructose diet (HFHFD) (Research Diets Inc., USA) or a normal chow diet, in which lights-on was referred to as ZT0/24 and lights-off as ZT12. RT-qPCR and western immunoblotting analyses were executed to identify the genotypes of the animals.

Some of the C57BL/6J mice were randomly allocated to four groups: ad libitum access to HFHFD (FA) (*n* = 8), access from ZT13 to ZT23 to an HFHFD (FT) (*n* = 10), ad libitum access to a chow diet (NA) (*n* = 10), and access from ZT13 to ZT23 to a chow diet (NT) (*n* = 10). Another group of C57BL/6J mice was used to identify the role of ferroptosis and was randomized into two subgroups: ad libitum access to HFHFD accompanied by liproxstatin-1 (Lip-1) (MedChemExpress, USA) injection (FA+Lip-1) and mice provided the appropriate vehicle (FA+Veh) for the last four weeks. Lip-1 was dissolved in PBS with a small amount of DMSO and administered via intraperitoneal (ip) injection to mice at a dosage of 10 mg per kg per day [27, 28]. The Per2^△hep^ and Per2^fl/fl^ mice were fed an HFHFD ad libitum from the beginning of the experiment.

### 2.2. Sample Collection

After 16 weeks of HFHFD or normal chow diet feeding, the mice were fasted from ZT0 and sacrificed at ZT8 to ZT10, and blood and liver tissue were collected. The blood was stored in a refrigerator at 4°C for 12 h. After centrifugation at 3000 rpm for 10–15 min, the serum was collected and stored at −80°C for further analyses. The liver tissues obtained from the mice were cut into small pieces and immediately frozen in liquid nitrogen and stored at −80°C for further analyses.

### 2.3. Glucose Tolerance Testing

For mice in the FA+Lip-1 and FA+Veh groups, we adopted an ip glucose tolerance test (IPGTT) [29, 30] the day before the mice were sacrificed to fully reflect the influence of Lip-1 on glucose metabolism. For the other mice, the IPGTT was performed at week 12 of the experiment. All mice were fasted for 10 h (from ZT23 to ZT9), and the IPGTT was conducted as follows according to a previous study [30]. We retrieved a second drop of blood from the tails, measured glucose concentrations (0 min) with a Portable Blood Glucose Meter (Johnson & Johnson, USA), and simultaneously injected a glucose solution (20%, 2 g per kg) ip into the mice. We then measured glucose concentrations using the same method as above at 30, 60, 90, and 120 min after glucose injection. The mice were fasted during the experiment but had free access to water.

### 2.4. Analysis of Serum Biochemical Indices

Serum levels of alanine aminotransferase (ALT), aspartate aminotransferase (AST), total cholesterol (TC), triglycerides (TGs), free fatty acids (FFAs), and iron were determined by commercial kits (Nanjing Jiancheng Bioengineering Institute, China) following the manufacturer's protocols.

### 2.5. Analysis of Liver Biochemical Indices

We removed the liver pieces that were frozen at −80°C and generated a liver homogenate (10%). The homogenate was centrifuged to obtain the supernatant, and the liver concentrations of TC, TG, FFA, iron, malondialdehyde (MDA), and glutathione (GSH) were assessed with commercial kits (Nanjing Jiancheng Bioengineering Institute, China) following the manufacturer's protocols. The data were then normalized to protein concentration.

### 2.6. Pathologic Examination of the Liver

Liver samples were fixed in 4% paraformaldehyde for 24 h, paraffin-embedded, sectioned at 5 *μ*M, and stained with hematoxylin and eosin (H&E) (Baso, China) and Sirius red (Solarbio, China). Other portions of the samples were fixed in optimal cutting temperature compound (OCT) (SAKURA, Japan) and frozen immediately in liquid nitrogen. After cutting the samples at 10 *μ*M, oil red O (Solarbio, China) staining was used to assess steatosis. All slices noted above were observed under an Olympus optical fluorescence microscope (Olympus Corporation, Japan).

### 2.7. Scoring of NAFLD Activity Score (NAS) and Fibrotic Stage

The NAS score (which ranged from 0 to 8) was calculated according to a previous study [31, 32] upon evaluation of H&E-stained sections; and the sum of the scores for steatosis (0-3), lobular inflammation (0-3), and hepatocyte ballooning (0-2) was considered the NAS score, with a score ≥ 5 strongly indicating NASH. The fibrotic stage was also evaluated according to a previous study and ranged from 0 to 4 [31].

### 2.8. RNA Sequencing

RNA-Seq experiments and data analysis were performed using fresh-frozen liver tissues from the FA+Veh and FA+Lip-1 mice (*n* = 3 per group) or Per2^△hep^ and Per2^fl/fl^ mice (*n* = 3 − 4 per group). After total RNA was extracted and quantified, RNA sequencing was implemented using an Illumina Novaseq 6000 sequencer [33] to obtain the raw data.

To ensure the accuracy of subsequent biological information analysis, the raw sequenced data were filtered by removing the linker sequence, low-quality reads, and reads with N to obtain clean data for subsequent analysis. The clean data were mapped to the genotype of *Mus musculus* to obtain comprehensive transcript information using STRA software (version 2.5.3a) [34]. We employed FeatureCounts [35] to retrieve the reads that mapped to the exonic regions and calculated the reads per kilobase of transcript per million reads mapped (RPKMs) [36] with the edgeR package (version 3.12.1) [37] to obtain the expression levels of the differential genes (DEGs).

The DEGs were identified with an absolute value of logFC > 1 and a *P* value < 0.05. A gene expression heatmap was obtained by the hierarchical clustering method, and Kyoto Encyclopedia of Genes and Genomes (KEGG) enrichment analysis and Gene Ontology (GO) analysis were applied using KOBAS software (version: 2.1.1) [38] to attain the biological meaning and function of the genes. A *P* value < 0.05 indicated statistically significant enrichment. RNA-Seq experiments and data analyses were performed by the Wuhan Seqhealth Technology Co., Ltd.

### 2.9. Transmission Electron Microscopy (TEM)

The examination of electron microscopic images was as previously reported [20]. Fresh liver sections were fixed in 2.5% glutaraldehyde (Servicebio, China) at 4°C for four hours, washed three times with phosphate-buffered saline (PBS, Servicebio, China) for 15 min each, and subsequently fixed with 1% OsO_4_ in 0.1 mol/L PBS at room temperature for two hours. After dehydration and penetration, the liver sections were embedded in epoxy resin. Thin slices were cut on an ultramicrotome (Leica, Germany) and stained with lead citrate and 2% uranyl acetate, and TEM was used to procure images.

### 2.10. Fluorescence Staining

Frozen sections of liver sections were rewarmed and subsequently fixed in 4% paraformaldehyde. After washing in PBS, 0.2% Triton X-100 was used for 10 min, followed by 10 min of PBS washing. The sections were then incubated with C11-Bodipy (5 *μ*mol/L, Invitrogen, USA) at 37°C for 30 min and washed in PBS [20]. Finally, the cellular nuclei were stained with 4′,6-diamidino-2′-phenylindole (DAPI, Servicebio, China) for 10 min, and images were observed with an Olympus optical fluorescence microscope (Olympus Corporation, Japan).

### 2.11. Real-Time Polymerase Chain Reaction (RT-qPCR) and Western Immunoblot Analysis

After total RNA was extracted and reverse-transcribed to cDNA, we performed RT-qPCR, and the fold change in mRNA expression was calculated with the 2^−*ΔΔ*Ct^ method [39] and compared using Student's *t* tests (the primers are depicted in Table 1). Total tissue proteins were extracted and denatured, and a PG-112 PAGE gel (10%, Epizyme Biomedical Technology Co., Ltd., China) was exploited separate the proteins. A PVDF membrane (Merck Millipore Ltd., USA) was adopted to transfer the proteins and was cut into pieces according to the molecular weights of the target proteins. We incubated membranes with the primary antibodies against Per2 rabbit mAb (ABclonal, China), Acsl4 rabbit mAb (ABclonal, China), Gpx4 mouse mAb (Proteintech, USA), Tfr1 rabbit pAb (ABclonal, China), PPAR*α* mouse mAb (ABclonal, China), and PPAR*γ* rabbit PAb (ABclonal, China) and utilized the secondary antibodies (AntGene, China) to detect the primary antibodies. The target proteins were ultimately visualized using a chemiluminescence kit (Vazyme, China).

### 2.12. Statistical Analysis

All experimental data are presented as mean ± standard error, and the data were analyzed with IBM SPSS 23. We adopted a one-way analysis of variance (ANOVA) and post hoc testing with least significant difference (LSD) or Dunnett's T3 tests (according to the homogeneity of variance observed) to identify differences among three or more independent groups [40, 41]. An independent sample *t* test was executed to determine the differences between the two independent groups. The figures in this experiment were created using GraphPad Prism 6.0 software. A *P* value < 0.05 indicated statistically significant differences.

## 3. Results

### 3.1. TRF Alleviates HFHFD-Induced NASH

We fed C57/BL6 J mice an HFHFD for 16 weeks to formulate a NASH model (the FA group), and a normal chow diet was used for the controls (NA group). To explore the effectiveness of TRF in the treatment of NASH, we fed TRF-treated mice with HFHFD for 10 h (from ZT13 to ZT23) per day (FT groups), with the FA group serving as a control. At last, we measured metabolic indices, liver injury markers, and liver pathologic findings of mice in the NA, NT, FA, and FT groups.

**Figure 1:**
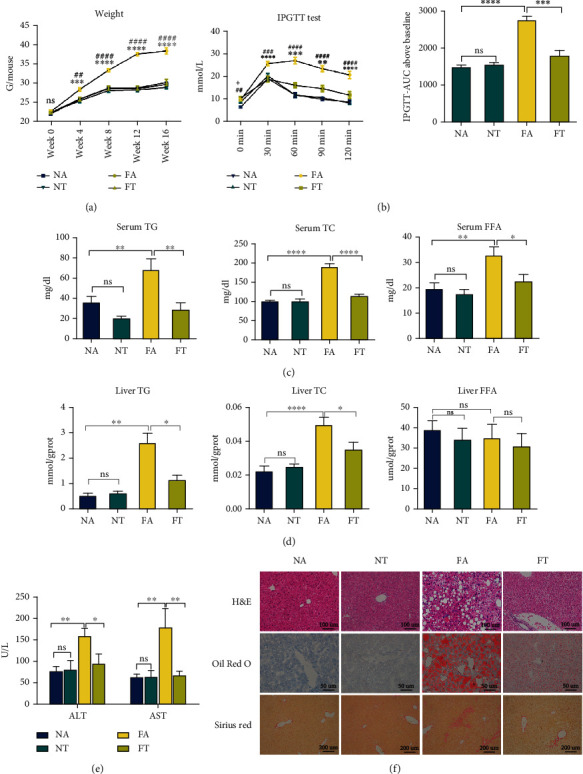
TRF alleviates HFHFD-induced NASH. (a) Body weights of mice measured at 0, 4, 8, 12, and 16 weeks in the present experiment; ^∗^FA vs. FT and ^#^FA vs. NA. (b) An IPGTT test was performed at the end of 12 weeks, and the corresponding area under the curve was calculated to assess differences among the four groups; ^∗^FA vs. FT and ^#^FA vs. NA. (c, d) Serum and liver TG, TC, and FFA levels are depicted to demonstrate lipid concentrations in the various groups. (e) Serum ALT and AST were measured to evaluate the levels of liver injury. (f) Pathologic findings in liver sections. Liver paraffin sections were stained with H&E (original magnification, 200x; scale bar, 100 *μ*m), oil red O (original magnification, 400x; scale bar, 50 *μ*m), and Sirius red (original magnification, 100x; scale bar, 200 *μ*m) to determine the levels of steatosis, inflammation, and fibrosis. Data are presented as mean ± SEM. ^∗^*P* < 0.05, ^∗∗^*P* < 0.01, ^∗∗∗^*P* < 0.001, ^∗∗∗∗^*P* < 0.0001, ^##^*P* < 0.01, ^###^*P* < 0.001, and ^####^*P* < 0.0001; ns: not significant (also refer to Figure S1).

We found that mice in the FA group developed NASH with significant weight gain; high serum glucose; high concentrations of TG, TC, and FFA in their serum; and high concentrations of TG and TC in their livers (Figures 1(a)–1(d)). Regarding liver injury markers, the levels of ALT and AST were also increased in the FA group (Figure 1(e)). Compared with the FA group, the TRF strategy significantly lowered the body weight; reduced the serum levels of TG, TC, and FFA and liver concentrations of TG and TC; decreased the ALT and AST levels; and improved the glucose tolerance without affecting food intake (Figures 1(a)–1(e) and S1A).

In addition to the improvement in liver enzymes and metabolic indices in the NASH mice, TRF also significantly alleviated the liver injury induced by HFHFD. Our results revealed that mice in the FA group developed significant steatosis, inflammation, and fibrosis with a higher NAS score and that these changes were significantly reduced in the FT group (Figures 1(f) and S1B–C). Furthermore, RT-qPCR showed that the expression of tumor necrosis factor- (Tnf-) *α* was elevated in the FA group and reduced in the FT group, confirming a reduction in overall inflammation (Figure S1D). These data showed that TRF effectively attenuated NASH with an improvement in pathologic findings, liver-injury markers, and metabolic indices.

### 3.2. TRF Reverses the Overexpression of the Circadian Gene Per2 in NASH Mice

**Figure 2:**
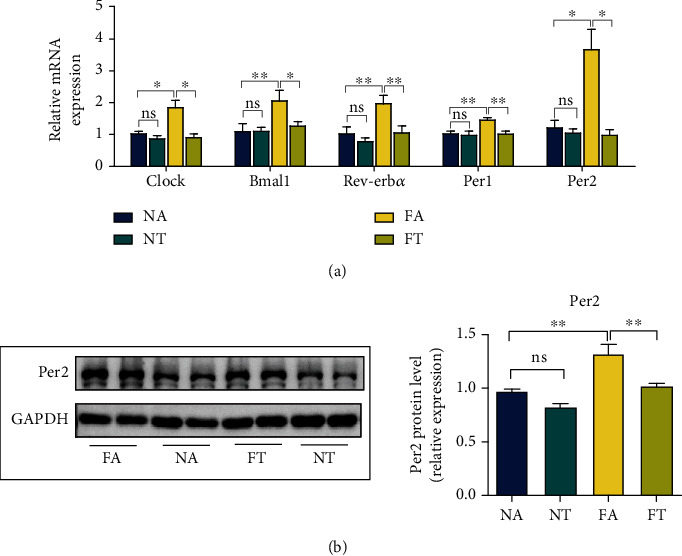
TRF reverses the overexpression of the circadian gene Per2 in NASH mice. (a) Hepatic mRNA levels for Bmal1, Clock, Per2, Per1, and Rev-Erb*α* were measured by RT-qPCR with GAPDH used as a control. (b) The Per2 protein level was measured by western blotting, and GAPDH was used as a control. Data are presented as mean ± SEM. ^∗^*P* < 0.05 and ^∗∗^*P* < 0.01; ns: not significant.

Earlier studies reported that the effect of TRF in metabolic disorders might be achieved by restoring the expression of circadian genes [6]. To verify this hypothesis, we implemented RT-qPCR and measured the expression of circadian rhythm-related genes. Our results showed that the expression of circadian genes such as Bmal1, Clock, Rev-Erb*α*, Per2, and Per1 increased in the FA group, and the strategy with TRF reduced the expression of the aforementioned circadian genes (Figure 2(a)).

Per2 is an indispensable gene in the modulation of circadian rhythm [13, 14] and participates in lipid and glucose metabolism [15, 16], thus attracting our attention. We therefore analyzed Per2 protein levels to identify the relationship between TRF and the Per2 gene. By applying western blotting, we ascertained that the protein levels of Per2 were elevated in the FA group and that TRF effectively inhibited the expression of the Per2 gene (Figure 2(b)).

### 3.3. TRF Suppresses the Ferroptosis in NASH Mice

Ferroptosis is reported to play an important role in the pathogenesis of NASH [20, 21]. Since TRF alleviated NASH, we intended to explore the role of TRF in ferroptosis. As we noted above, the ferroptosis is characterized by the accumulation of LPO and iron [17], alterations in mitochondrial morphology, and an elevated expression of some ferroptosis-related genes. We therefore examined the following components.

**Figure 3:**
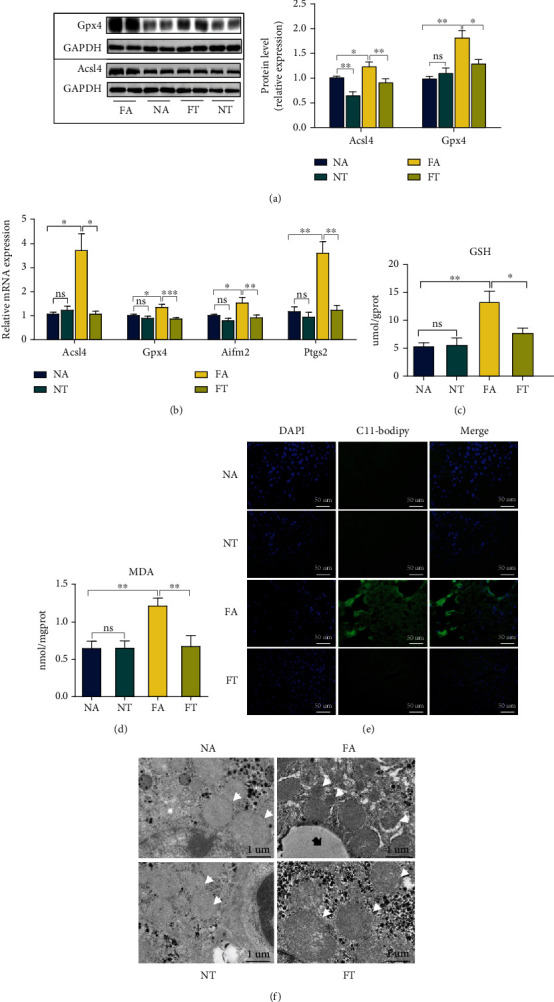
TRF suppresses the ferroptosis in NASH mice. (a) Acsl4 and Gpx4 protein levels were measured by western blotting, with GAPDH used as a control. (b) Hepatic mRNA levels for Acsl4, Gpx4, Aifm2, and Ptgs2 were measured by RT-qPCR; and GAPDH was used as a control. (c, d) Liver GSH and MDA concentrations were quantified with commercial kits. (e) Fluorescence staining showing lipid peroxidation (LPO) levels in the FA, FT, NA, and NT groups. The nuclei were labeled with DAPI (left), and LPO (middle) was labeled with C11-Bodipy (original magnification, 400x; scale bar, 50 *μ*m). (f) Electron microscopic analysis of the morphologic changes in mitochondria; the white arrow indicates lipid droplets, and the black arrow indicates mitochondria (original magnification, 5000x; scale bar, 1 *μ*m) (NA and NT, *n* = 2; FA and FT, *n* = 3). ^∗^*P* < 0.05, ^∗∗^*P* < 0.01, and ^∗∗∗^*P* < 0.001; ns: not significant.

First, we measured the expression of ferroptosis-related genes. The mRNA levels for anti-acyl-CoA synthetase long-chain family member 4 (Acsl4), glutathione peroxidase 4 (Gpx4), apoptosis-inducing factor mitochondria-associated 2 (Aifm2), and prostaglandin-endoperoxide synthase 2 (Ptgs2); the protein levels of Acsl4 and Gpx4; and the liver GSH concentrations were upregulated in the FA group but reduced by the TRF strategy (Figures 3(a)–3(c)). Second, the levels of LPO in the FA group that we measured by a combination of the specific fluorescent probe C11-Bodipy (581/591) with the concentration of MDA [42] were augmented, and electron microscopy revealed shrunken mitochondria and increased mitochondrial membrane density; TRF then subsequently relieved both of these indices (Figures 3(d)–3(f)). These results showed that ferroptosis occurred in NASH and that TRF inhibited ferroptosis.

### 3.4. TRF Reduces Iron Accumulation in Serum and Liver after Induction by HFHFD

**Figure 4:**
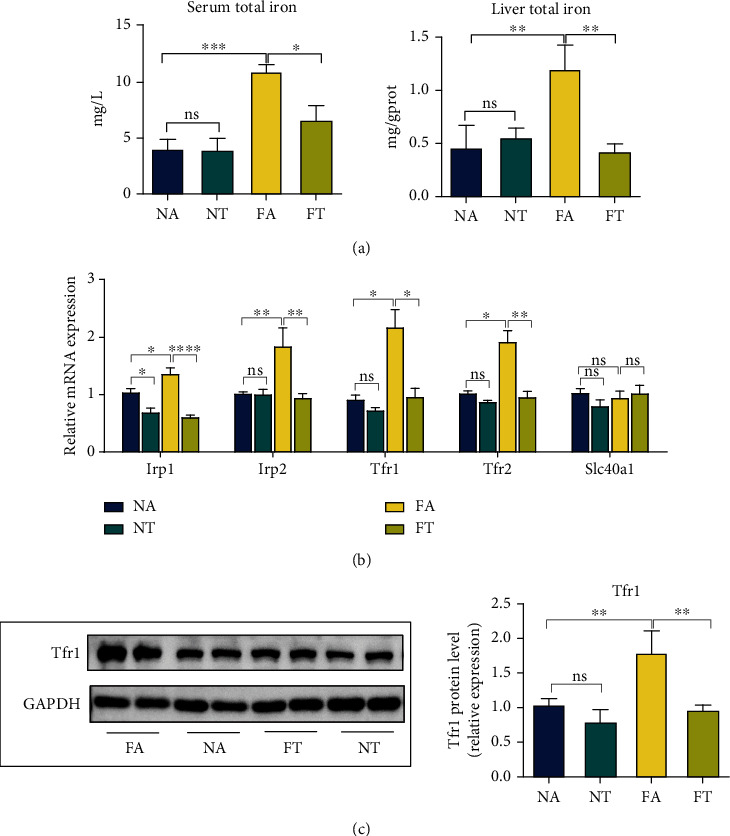
TRF reduces the iron accumulation in the serum and liver as induced by HFHFD. (a) Serum and liver iron concentrations were determined using a commercial kit to compare the iron overload levels among the four groups. (b) Hepatic mRNA levels for Irp1, Irp2, Tfr1, Tfr2, and Slc40a1 were measured by RT-qPCR, and GAPDH was used as a control. (c) Tfr1 protein levels were measured by western blotting, with GAPDH used as a control. Data are presented as mean ± SEM. ^∗^*P* < 0.05, ^∗∗^*P* < 0.01, ^∗∗∗^*P* < 0.001, and ^∗∗∗∗^*P* < 0.0001; ns: not significant.

Investigators have reported iron overload in both animal and clinical studies [43, 44], promoting NASH and ferroptosis to a significant degree [45, 46]. Consistent with these studies, we discerned that the serum and liver iron concentrations rose in the FA group and that the TRF strategy significantly reduced these concentrations (Figure 4(a)). Furthermore, when we determined the expression of the iron metabolism-related genes iron regulatory protein (Irp)1, Irp2, transferrin receptor (Tfr)1, Tfr2, and ferroportin (Slc40a1), we noted that the expression of Irp1, Irp2, Tfr1, and Tfr2 was enhanced in the FA group and attenuated in the FT group compared with the FA group (Figure 4(b)).

Tfr1 is a key receptor that transports iron to cells [47], and it is reported to contribute to ferroptosis [48, 49]. We thus analyzed Tfr1 protein levels and demonstrated that its expression was upregulated in the FA group and restored by TRF, verifying iron overload in NASH and its mitigation by TRF (Figure 4(c)).

### 3.5. Hepatocyte-Specific Knockout of Per2 Mitigates HFHFD-Induced NASH

**Figure 5:**
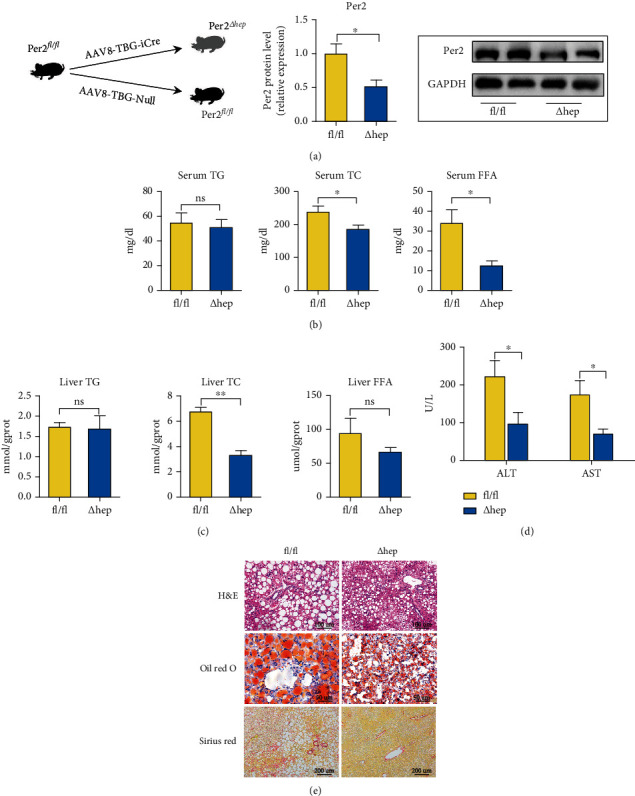
Hepatocyte-specific knockout of Per2 alleviates HFHFD-induced NASH. (a) Construction of the Per2^△hep^ mouse model and its verification by RT-qPCR and western blotting. (b, c) Serum and liver TG, TC, and FFA levels are depicted to demonstrate the lipid concentrations between the two groups. (d) ALT and AST levels were measured with a commercial kit to evaluate the levels of liver injury between the Per2^△hep^ and Per2^fl/fl^ mice. (e) Pathologic findings in the liver. Liver paraffin sections were stained with H&E (original magnification, 200x; scale bar, 100 *μ*m), oil red O (original magnification, 400x; scale bar, 50 *μ*m), and Sirius red (original magnification, 100x; scale bar, 200 *μ*m) to determine the levels of steatosis, inflammation, and fibrosis in Per2^△hep^ and Per2^fl/fl^ mice. Both Per2^△hep^ and Per2^fl/fl^ mice were fed with HFHFD to induce NASH. Data are presented as mean ± SEM. ^∗^*P* < 0.05 and ^∗∗^*P* < 0.01; ns: not significant (also refer to Figure S2).

The aforementioned information revealed that TRF not only ameliorated NASH but also suppressed ferroptosis, Per2 expression, and iron overload. To investigate how Per2 participated in NASH and whether Per2 regulated the occurrence of ferroptosis, we deployed the use of Per2^△hep^ mice (Figures 5(a) and S2A). In previous studies, Per2 knockout or inhibition improved glucose and lipid metabolism [15, 16]. In our study, we fed both Per2^△hep^ and Per2^fl/fl^ mice with an HFHFD to examine the effects of Per2 on NASH and found that fasting blood glucose (Figure S2B), serum levels of TC and FFA, and liver TC levels (Figures 5(b) and 5(c)) were lower in Per2^△hep^ mice, while body weight, liver/body weight ratio, and IPGTT test (Figures S2B-C) were unchanged between the two groups.

In addition to the metabolic indices, we also evaluated the serum levels of hepatic enzymes and the degree of liver injury, and our data showed that both ALT and AST levels were lower in Per2^△hep^ mice than in Per2^fl/fl^ mice (Figure 5(d)). Furthermore, our pathologic findings depicted Per2^△hep^ mice as exhibiting alleviated steatosis, inflammatory cell infiltration, and fibrosis with NASH (Figures 5(e) and S2D–E). RT-qPCR was used to assess inflammation in the liver, and these results characterized expression levels of interleukin- (IL-) 6 and IL-1*β* as diminished in Per2^△hep^ mice (Figure S2F). These results indicated that Per2 was not only elevated in HFHFD-induced NASH but that it also aggravated NASH.

### 3.6. Hepatocyte-Specific Knockout of Per2 May Mitigate NASH by Inhibiting Ferroptosis

**Figure 6:**
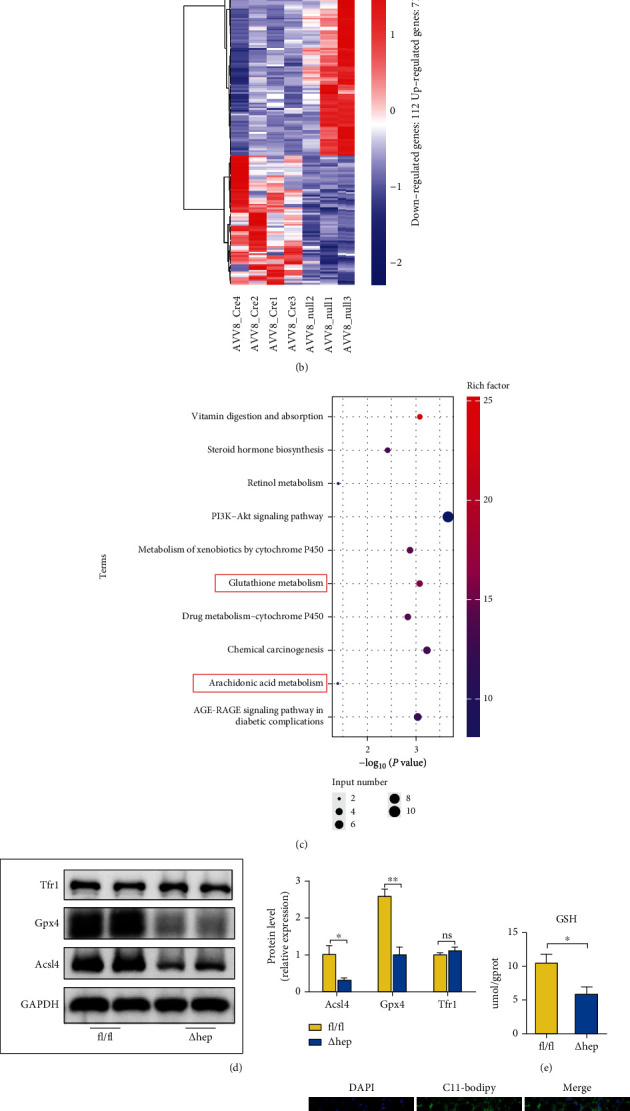
Hepatocyte-specific knockout of Per2 might alleviate NASH by inhibiting ferroptosis. (a) Volcano plot showing upregulated and downregulated genes in Per2^△hep^ and Per2^fl/fl^ mice. (b) Heatmap showing the differentially expressed genes (DEGs) between Per2^△hep^ and Per2^fl/fl^ mice; blue indicates low expression and red indicates high expression. (c) The DEGs between Per2^△hep^ and Per2^fl/fl^ mice were identified with the Kyoto Encyclopedia of Genes and Genomes (KEGG) pathway enrichment analysis. Rich factor (%) was the ratio of the number of DEGs annotated in a pathway (as indicated in the *y*-axis) to the number of all genes annotated in the pathway. (d) Acsl4, Gpx4, and Tfr1 protein levels were measured by western blotting, and GAPDH was used as a control. (e) Liver GSH concentrations were quantified by a commercial kit. (f) Electron microscopic evaluation of the morphologic changes in mitochondria; the white arrow indicates lipid droplets and the black arrow indicates mitochondria (original magnification, 5000x; scale bar, 1 *μ*m) (Per2^fl/fl^, *n* = 2; Per2^△hep^, *n* = 3). (g) Fluorescence staining represents lipid peroxidation (LPO) levels in the livers of Per2^△hep^ and Per2^fl/fl^ mice. The nuclei were labeled with DAPI (left), and LPO (middle) was labeled by C11-Bodipy (original magnification, 400x; scale bar, 50 *μ*m). (h) Liver MDA concentrations were quantified with a commercial kit. (i) Serum and liver iron concentrations were determined using a commercial kit to compare the iron-overload levels. Both Per2^△hep^ and Per2^fl/fl^ mice were fed with HFHFD to induce NASH. Data are presented as mean ± SEM. ^∗^*P* < 0.05 and ^∗∗^*P* < 0.01; ns: not significant (also refer to Figure S2).

We conducted RNA-Seq to explore the possible mechanisms underlying the alleviation of NASH by hepatocyte-specific knockout of Per2 and demonstrated that this knockout significantly promoted the expression of 71 genes and reduced the expression of 112 genes (Figures 6(a) and 6(b)). Furthermore, the KEGG analysis showed that the DEGs perturbed by Per2 were principally enriched in the arachidonic acid metabolism pathway and glutathione metabolism pathway (Figure 6(c)). Ferroptosis can be induced by the peroxidation of polyunsaturated fatty acids (PUFAs) and can be inhibited by the GSH and GPX4 pathways [50, 51]. Arachidonic acid is a type of PUFAs, and its metabolic regulation has been suggested to influence ferroptotic sensitivity [52]. Therefore, the DEGs enriched in the arachidonic acid and glutathione metabolism pathways implied that hepatocyte-specific knockout of Per2 alleviated NASH by regulating ferroptosis.

To verify the hypothesis we mentioned above, we measured the expression of ferroptosis-related genes. Our results indicated that the expression of Acsl4, Gpx4, Aifm2, and Ptgs2 was downregulated in Per2^△hep^ mice (Figure S2G) and that Acsl4 and Gpx4 protein levels and liver GSH levels were attenuated (Figures 6(d) and 6(e)). Furthermore, when we assessed LPO levels and mitochondrial morphologic changes in Per2^△hep^ and Per2^fl/fl^ mice, we found that the levels of LPO as reflected by C11-Bodipy and MDA concentration were reduced in Per2^△hep^ mice and that mitochondrial morphology was restored (Figures 6(f)–6(h)), confirming that Per2 promoted ferroptosis. Our results with regard to iron metabolism revealed that Per2 did not influence either serum or liver iron levels and that the expression of Tfr1 was also not altered by Per2 (Figures 6(d) and 6(i) and S2H).

### 3.7. Ferroptosis Occurs in HFHFD-Induced NASH and Promotes Its Progression

**Figure 7:**
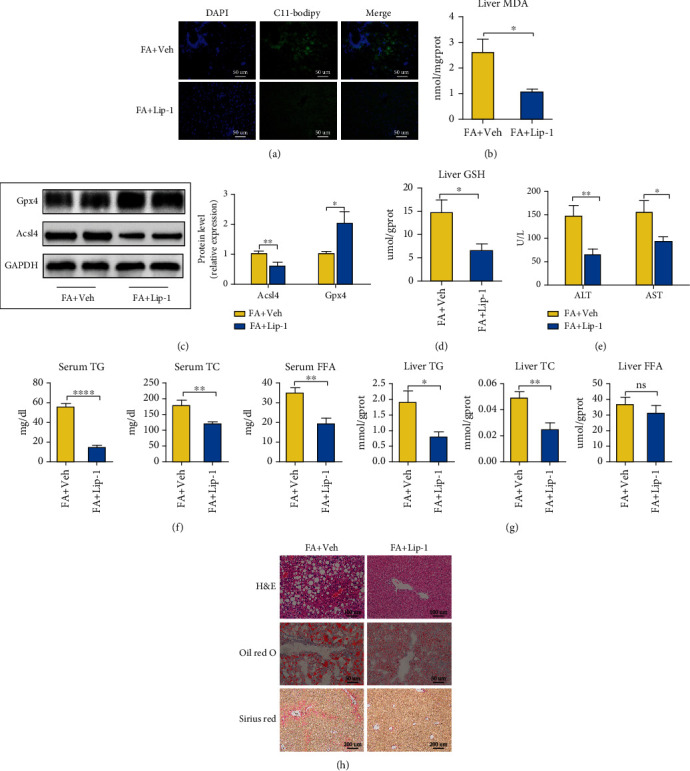
Ferroptosis occurs in HFHFD-induced NASH and promotes the progression of NASH. (a) Fluorescence staining showing lipid peroxidation (LPO) levels in the livers of the FA+Veh and FA+Lip-1 groups. The nuclei were labeled with DAPI (left) and LPO (middle) was labeled by C11-Bodipy (original magnification, 400x; scale bar, 50 *μ*m). (b) Liver MDA concentrations were measured with a commercial kit. (c) Acsl4 and Gpx4 protein levels were measured with western blotting, with GAPDH used as a control. (d) Liver GSH concentrations were measured by a commercial kit. (e) ALT and AST levels were determined by commercial kits to evaluate the levels of liver injury. (f, g) Serum and liver TG, TC, and FFA levels are depicted to demonstrate the lipid concentrations between the two groups. (h) Pathologic findings in liver sections. Liver paraffin sections were stained with H&E (original magnification, 200x; scale bar, 100 *μ*m), oil red O (original magnification, 400x; scale bar, 50 *μ*m), and Sirius red (original magnification, 100x; scale bar, 200 *μ*m) to determine the levels of steatosis, inflammation, and fibrosis in the FA+Veh and FA+Lip-1 groups. Data are presented as mean ± SEM. ^∗^*P* < 0.05, ^∗∗^*P* < 0.01, and ^∗∗∗∗^*P* < 0.0001; ns: not significant (also refer to Figure S3).

To identify a role for ferroptosis in NASH, we applied liproxstatin-1 (Lip-1) to suppress ferroptosis in mice. Consistent with a previous study [20], the levels of LPO in the liver of Lip-1 treated mice (FA+Lip-1) as reflected by C11-Bodipy and MDA concentrations were reduced relative to those in the control mice (FA+Veh) (Figures 7(a) and 7(b)). Furthermore, RT-qPCR assay showed that the expression of Acsl4 and Ptgs2 was reduced, and that of Gpx4 and Aifm2 was increased in the FA+Lip-1 group (Figure S3A). Western blotting indicated that Acsl4 protein levels as well as the liver GSH concentrations were reduced and that Gpx4 protein levels were enhanced in the FA+Lip-1 group (Figures 7(c) and 7(d)). These results suggested the presence of ferroptosis in HFHFD-induced NASH.

In addition, body weights declined significantly, with a diminution in the liver/body weight ratio, but without a reduction in food intake in the group provided Lip-1 (Figures S3B–C). ALT and AST levels were also reduced as were serum concentrations of TG, TC, and FFA and liver concentrations of TG and TC with Lip-1 treatment (Figures 7(e)–7(g)). With respect to pathologic findings, Lip-1 treatment significantly reduced steatosis, inflammatory cell infiltration, and fibrosis (Figure 7(h)) and produced a lower NAS score (Figures S3D–E). These results indicate that ferroptosis is critical to the progression of NASH. However, Lip-1 exerts a limited influence on glucose metabolism (Figure S3F).

### 3.8. Ferroptotic Participation in NASH May Be Related to the Inhibition of PPAR*α*

**Figure 8:**
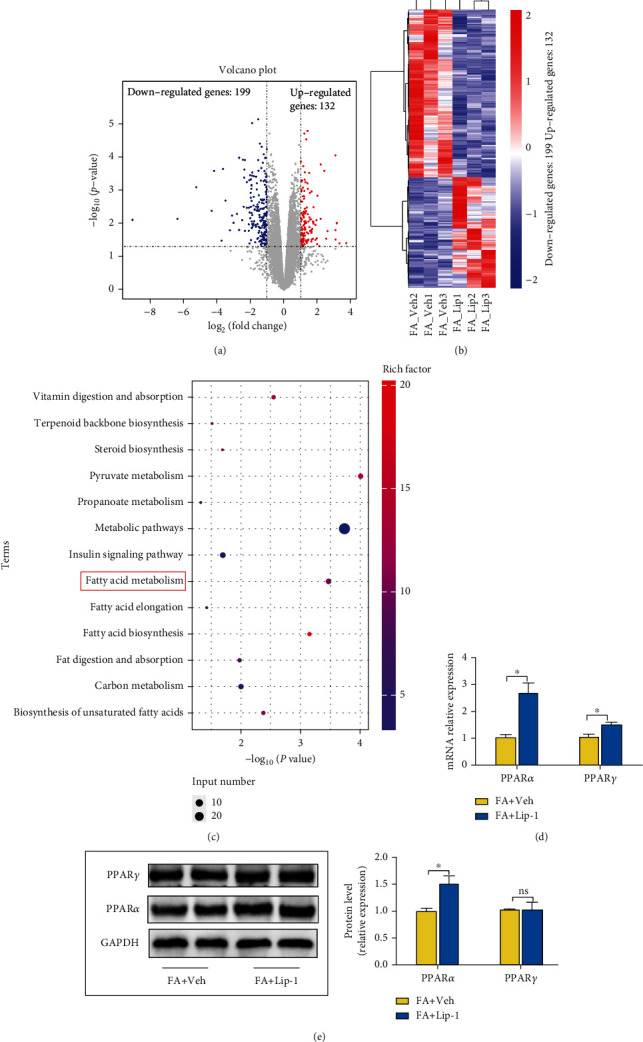
Ferroptotic participation in NASH might be related to inhibition of PPAR*α*. (a) Volcano plot showing the upregulated and downregulated genes in the Lip-1 treatment group. (b) Heatmap shows the DEGs between the FA+Veh and FA+Lip-1 groups; blue indicates low expression and red indicates high expression. (c) The DEGs between the FA+Veh and FA+Lip-1 groups were identified with the Kyoto Encyclopedia of Genes and Genomes (KEGG) pathway enrichment analysis. Rich factor (%) was the ratio of the number of DEGs annotated in a pathway (as indicated in the *y*-axis) to the number of all genes annotated in the pathway. (d) Hepatic mRNA levels for PPAR*α* and PPAR*γ* were measured by RT-qPCR, and GAPDH was used as a control. (e) PPAR*α* and PPAR*γ* protein levels in the FA+Veh and FA+Lip-1 groups were assessed by western blotting and GAPDH was used as a control. Data are presented as mean ± SEM. ^∗^*P* < 0.05; ns: not significant.

When we executed RNA-Seq to determine the mechanism by which ferroptosis participated in NASH, we uncovered 132 upregulated and 199 downregulated genes in the FA+Lip-1 group (Figures 8(a) and 8(b)). KEGG pathway enrichment analysis showed that the DEGs were mainly enriched in the fatty acid metabolism pathway between the FA+Veh and FA+Lip-1 groups (Figure 8(c)).

The family of peroxisome proliferator-activated receptors (PPARs) regulates energy metabolism, and PPAR*α* and PPAR*γ* are primarily expressed in the liver of NASH [24, 53–55]. We therefore measured PPAR*α* and PPAR*γ* mRNA and protein levels by RT-qPCR and western blotting and demonstrated that the expression of PPAR*α* was upregulated in the FA+Lip-1 group, but that protein levels of PPAR*γ* were unaltered (Figures 8(d) and 8(e)). These results suggested that ferroptosis participated in NASH by regulating the expression of PPAR*α*.

### 3.9. TRF and Per2 Knockout Promote the Expression of PPAR*α*

**Figure 9:**
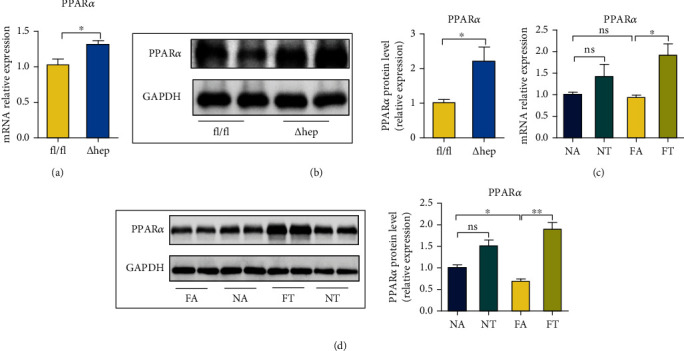
TRF and Per2 knockout promote the expression of PPAR*α*. (a) Hepatic mRNA levels for PPAR*α* in Per2^△hep^ and Per2^fl/fl^ mice were measured by RT-qPCR, and GAPDH was used as a control. (b) PPAR*α* protein levels in Per2^△hep^ and Per2^fl/fl^ mice were measured using western blotting, with GAPDH used as a control. (c) Hepatic mRNA levels for PPAR*α* in the NA, NT, FA, and FA groups were measured by RT-qPCR, and GAPDH was used as a control. (d) PPAR*α* protein levels in the NA, NT, FA, and FA groups were analyzed by western blotting, with GAPDH used as a control. Both Per2^△hep^ and Per2^fl/fl^ mice were fed with HFHFD to induce NASH. Data are presented as mean ± SEM. ^∗^*P* < 0.05; ns: not significant.

We analyzed the mRNA and protein levels for PPAR*α* in both TRF-treated and Per2^△hep^ mice and demonstrated that hepatocyte-specific knockout of Per2 significantly augmented the expression of PPAR*α* (Figures 9(a) and 9(b)) and that TRF also elevated the expression of PPAR*α* (Figures 9(c) and 9(d)). These data indicated that PPAR*α* might constitute a target in the process by which TRF alleviated NASH.

## 4. Discussion

We herein examined the effectiveness and underlying mechanisms of action of TRF in the treatment of NASH, uncovering a pivotal role for the circadian gene Per2 and ferroptosis in the pathogenesis and progression of NASH. We also hypothesized that Per2 participated in NASH by promoting ferroptosis and inhibiting the expression of PPAR*α*.

Authors of previous studies have reported a variety of modeling methods for NASH [56]. In this investigation, we chose an HFHFD to construct a NASH model in which corn starch and maltodextrin were replaced by fructose. As many foods rich in fructose are consumed in modern Western societies [57], the HFHFD-induced NASH model was thereby shown to be more similar to human NASH, and exhibiting more serious inflammation, fibrosis, and oxidative stress [56–58] relative to other dietary modeling methods. In a study in which the authors compared the animal models with human NAFLD, HFHFD-induced NAFLD exerted the greatest similarity to NAFLD in humans in both metabolic phenotype and histology; and HFHFD alone or combined with ip carbon tetrachloride (CCl4) injection mimicked NAFLD in humans with respect to gene expression [59]. Although an HFHFD emulates human NAFLD, there remains some limitations. First of all, no model duplicates all the features of NAFLD in metabolic phenotype and histology due to the complicated pathogenesis of human NAFLD, and the high proportion of HCC and lack of significant atherosclerosis in HFHFD-induced mouse models do not at all resemble human NAFLD [60, 61]. Second, as previous studies have revealed, an HFHFD necessitates an extensive period to induce cirrhosis and hepatocellular carcinoma (HCC) [59]. We herein thus aimed to explore novel underlying pathogenesis of human NASH, establishing a mouse model that is extremely similar in metabolic phenotype, histology, and gene expression, thereby increasing the reliability of our results to a considerable extent.

NASH is a type of progressive disease that occurs in both children and adults, and the characteristics of which are excessive fat accumulation and inflammatory cell infiltration in the liver [1, 2]. Fat accumulation is not only the basic characteristic of NASH but also promotes chronic inflammation and the progression of NASH through lipotoxicity [62]. In the present study, we ascertained that the TRF strategy, hepatocyte-specific knockout of Per2, and the application of a ferroptotic inhibitor not only improved metabolic indices and liver enzymes but also reduced liver steatosis, inflammation, and fibrosis induced by HFHFD. These results indicated that TRF effectively alleviated NASH and that both Per2 and ferroptosis participated in the pathogenesis of NASH.

Per2 is an important circadian gene. Mice who lost Per2 gene function suffered serious liver injury when exposed to CCl4 [63], emphasizing the protective role of Per2. However, other authors reported that mice lacking the Per2 gene or functional Per2 protein manifested an improvement in some metabolic indices [15, 16], suggesting that Per2 was an aggravating factor in metabolism-related disease. We herein first generated Per2^△hep^ mice and found that Per2 knockout significantly alleviated NASH, confirming that Per2 was a factor that exacerbated NASH. It should be noted that this was the first-ever study designed to explore the role of Per2 in NASH by using Per2^△hep^ mice. Unlike the traditional knockout mice, the use of Per2^△hep^ mice principally focuses on the functional exploration of target genes in specific organs, thus enabling a more precise hepatic role for Per2.

While ferroptosis is a newly discovered type of cell death that has already been investigated in many diseases, its determination is difficult. The optimal detection methods currently comprise the measurements of LPO accumulation, changes in mitochondrial morphology, overexpression of some ferroptosis-related genes, and the efficacy of ferroptotic inhibitors [20, 21]. By using the aforementioned methods, we thus initially confirmed that ferroptosis occurred in HFHFD-induced NASH.

To explore the mechanism by which TRF alleviated NASH, we discerned that TRF not only inhibited the expression of Per2 but also significantly inhibited ferroptosis—with reduced LPO levels, attenuated expression of ferroptosis-related genes, and the remission of morphologic changes in mitochondria. According to our RNA-Seq results, we found that Per2 is related to the occurrence of ferroptosis, and additional experiments indicated that ferroptosis was inhibited in Per2^△hep^ mice. All of these results indicated that TRF inhibited the expression of the Per2 gene and that the amelioration of NASH by hepatocyte-specific knockout of Per2 might be related to the inhibition of ferroptosis. To our knowledge, this was the first study to examine a possible relationship between Per2 and ferroptosis.

GSH and Gpx4 are notable antioxidants in cells, and in the presence of GSH, Gpx4 plays a protective role in the process of ferroptosis by reducing the levels of LPO [64]; therefore, ferroptosis is caused by either GSH or Gpx4 depletion [65, 66]. However, in some cases, their expression increases, and GSH and Gpx4 then act as protective antioxidants in the process of ferroptosis [20, 28]; therefore, the elevated expression of Gpx4 and GSH also indicate the onset of ferroptosis. Consistent with previous reports [20, 28], this study showed that Gpx4 and GSH expression rates were elevated in HFHFD-induced NASH and that both TRF and hepatocyte-specific knockout of Per2 restored their expression. Congruent with a previous study, the expression of Gpx4 was elevated in a FA+Lip-1 group [67]; but in contradistinction, GSH concentration decreased in the FA+Lip-1 group, which was not consistent with Gpx4. Lip-1 is a lipid autoxidation inhibitor that effectively inhibits ferroptosis, but the other effects of Lip-1 are arcane and require further investigation.

Excessive fat accumulation in the liver promotes the progression of NASH [68], and the mechanisms generating hepatic steatosis constitutes the focal point of NASH pathogenesis. Among the PPAR subtypes, PPAR*α* is primarily expressed in the liver, and PPAR*γ* is highly expressed in the liver of individuals with NASH [24, 53–55]. According to our RNA-Seq results, the DEGs were primarily enriched in the fatty acid metabolism pathway between the FA+Veh and FA+Lip-1 groups. Therefore, we hypothesized a relationship between ferroptosis and PPAR*α* or PPAR*γ*, and our data revealed that Lip-1 promoted the expression of PPAR*α* without affecting PPAR*γ*, while TRF and hepatocyte-specific knockout of Per2 both enhanced the expression of PPAR*α*. These results suggest that PPAR*α* might be the downstream mechanism governing ferroptosis in its aggravation of NASH. Investigators previously reported that PPAR*α* activation alleviated ferroptosis by exploiting PPAR*α* knockout mice and a PPAR*α* ligand [69] and that activation of PPAR*α* exerted similar effects in protecting against ferroptosis-induced liver injury [69]; this indicated to these authors that PPAR*α* might be the regulator of ferroptosis. In the current analysis, we found that the inhibition of ferroptosis promoted PPAR*α* expression, also implying that PPAR*α* was a component in the downstream mechanism underlying the process of ferroptosis in the exacerbation of NASH. Furthermore, although we are the first to raise this possibility, additional studies are needed to confirm this hypothesis.

In the present study, we found that TRF repressed the expression of Per2, that Per2 promoted ferroptosis, and that the expression of PPAR*α* was increased by TRF, hepatocyte-specific knockout of Per2, and the inhibition of ferroptosis. We therefore hypothesized that TRF likely alleviated NASH by regulating the circadian gene Per2 and ferroptosis and that this was ultimately related to the promotion of PPAR*α* expression. However, during our investigation we determined that hepatocyte-specific knockout of Per2 did not influence the body weight of mice, while TRF and Lip-1 did; and it is acknowledged that obesity itself is closely related to the prevalence and severity of NASH [70]. Therefore, in addition to the pathway we noted above, weight loss induced by TRF might constitute another means by which TRF alleviates NASH.

## 5. Limitations of the Study

For this study, we used an HFHFD to induce NASH, creating signs similar to those for human NASH; and we confirmed that TRF's alleviation of NASH was related to the regulation of the circadian gene Per2. Furthermore, we indicated an important role for ferroptosis in NASH and demonstrated that ferroptosis was regulated by Per2. The limitations to our study included the following. First, we did not examine the changes in the circadian rhythms for the various indicators—although this did not influence the conclusion that TRF and Per2 regulated the onset of ferroptosis. Second, as we used Per2^△hep^ mice to examine the role of Per2 in NASH (implementing a model in which all mice were fed with an HFHFD), the results we generated from the Per2^△hep^ mice did not modify the conclusions of this investigation; however, our conclusions may not apply to normal diets. Third, as the mechanism underlying NASH is complex and we only examined the effects of ferroptosis on the PPAR family, there might be additional pathways involved in the overall process. Finally, while we hypothesized that the PPAR*α* pathway was a downstream mechanism subserving ferroptosis in exacerbating NASH, we did not provide further verification. Although this should not affect the conclusions, additional studies are required to confirm these data. We posit that the conclusions of our study are reasonable, but also further elucidation is still needed.

## 6. Conclusions

In this study, we uncovered TRF as an effective therapeutic strategy for NASH. After further scrutiny, we also determined that the circadian gene Per2 might participate in NASH by promoting ferroptosis; moreover, ferroptosis likely promoted NASH by inhibiting the expression of PPAR*α*. Thus, these results fulfilled the pathophysiologic underpinning mechanisms of NASH and provided some potentially novel treatment targets for this disorder.

## Figures and Tables

**Table 1:** The primers used in this article.

Gene primers	Forward (5′-3′)	Reverse (5′-3′)
GAPDH	AACTTTGGCATTGTGGAAGG	ACACATTGGGGGTAGGAACA
Tnf-*α*	AGGGACCTCTCTCTAATCAG	TGGGAGTAGATGAGGTACAG
IL-6	GAAATGATGGATGCTACCAAACTG	GACTCTGGCTTTGTCTTTCTTGTT
IL-1*β*	TTGTTGATGTGCTGCTGTGA	TGTGAAATGCCACCTTTTGA
Per1	GGTTCAGGATCCCACGAAG	AAGAGTCGATGCTGCCAAAG
Per2	CACACTTGCCTCCGAAATAACTC	AGCGCACGGCTGTCTGA
Bmal1	AACCTTCCCGCAGCTAACAG	AGTCCTCTTTGGGCCACCTT
Clock	GGCGTTGTTGATTGGACTAGG	GAATGGAGTCTCCAACACCCA
Rev-erb*α*	TGGCCTCAGGCTTCCACTATG	CCGTTGCTTCTCTCTCTTGGG
Acsl4	CTCACCATTATATTGCTGCCTGT	TCTCTTTGCCATAGCGTTTTTCT
Gpx4	GCCTGGATAAGTACAGGGGTT	CATGCAGATCGACTAGCTGAG
Aifm2	TTACAAGCCAGAGACTGACCAA	ACAAGGCCTGTCACTGAAGAG
Ptgs2	TGAGCAACTATTCCAAACCAGC	GCACGTAGTCTTCGATCACTATC
Irp1	AGAACCCATTTGCACACCTTG	AGCGTCCGTATCTTGAGTCCT
Irp2	CTGCTATGAGGGAGGCAGTG	TGCAGGGAAGCTTCTTAGGC
Tfr1	TCCTGTCGCCCTATGTATCT	CGAAGCTTCAAGTTCTCCACTA
Tfr2	TTGGGGTCTACTTCGGAGAGT	GACAGGAGCCTAAGTGCTCAG
Slc40a1	ACCAAGGCAAGAGATCAAACC	AGACACTGCAAAGTGCCACAT

## Data Availability

All the data in this article are available from the corresponding author Xiaoli Pan. And the data of RNA-Seq are available in NCBI (BioProject: PRJNA836098 and PRJNA836019).

## Abbreviations

NASH: Nonalcoholic steatohepatitis
TRF: Time-restricted feeding
PPAR: Peroxisome proliferator activated receptor
NAFLD: Nonalcoholic fatty liver disease
LPO: Lipid peroxidation
Per2^△hep^: Hepatocyte-specific Per2-knockout
HFHFD: High-fat and high-fructose diet
FA: Ad libitum access to HFHFD
FT: From ZT13 to ZT23 access to HFHFD
NA: Ad libitum access to chow diet
NT: From ZT13 to ZT23 access to chow diet
Lip-1: Liproxstatin-1
Iv: Injected intravenously
IPGTT: Intraperitoneal glucose tolerance test
Ip: Intraperitoneal
ALT: Alanine aminotransferase
AST: Aspartate aminotransferase
TC: Total cholesterol
TG: Triglyceride
FFA: Free fatty acid
MDA: Malondialdehyde
GSH: Glutathione
H&E: Hematoxylin and eosin
OCT: Optimal cutting temperature compound
NAS: NAFLD activity score
RPKMs: Reads per kilobase of transcript per million reads mapped
DEGs: Differential genes
KEGG: Kyoto Encyclopedia of Genes and Genomes Enrichment
GO: Gene Ontology
PBS: Phosphate-buffered saline
TEM: Transmission electron microscopy
DAPI: 4′,6-Diamidino-2′-phenylindole
RT- qPCR: Real-time polymerase chain reaction
ANOVA: Analysis of variance
LSD: Least significant difference
Tnf: Tumor necrosis factor
Acsl4: Anti-acyl-CoA synthetase long-chain family member 4
Gpx4: Glutathione peroxidase 4
Aifm2: Apoptosis-inducing factor mitochondria-associated 2
Ptgs2: Prostaglandin-endoperoxide synthase 2
Irp: Iron regulatory protein
Tfr: Transferrin receptor
Slc40a1: Ferroportin
IL: Interleukin
CCL4: Carbon tetrachloride

## Supplementary Materials

Supplementary Materials Some detected indices of mice in FA, FT, NA, and NT groups, Per2^△hep^ and Per2^fl/fl^ mice, and FA+Veh and FA+Lip-1 groups. Figure S1: TRF alleviates HFHFD-induced NASH. Figure S2: hepatocyte-specific knockout of Per2 might alleviate NASH by inhibiting ferroptosis without influencing the iron concentration. Figure S3: ferroptosis occurs and participates in HFHFD-induced NASH.
