# Supplementary material for: Attenuation by Time-Restricted Feeding of High-Fat and High-Fructose Diet-Induced NASH in Mice Is Related to Per2 and Ferroptosis
Source: Oxid Med Cell Longev. 2022 Oct 15;2022:8063897. doi: 10.1155/2022/8063897 (PMC9588383; doi:10.1155/2022/8063897)

**Figure legends**

**Figure S1. TRF alleviates HFHFD-induced NASH**.

(A) The liver/body weight ratio and food intake were measured in FA, FT, NA and NT groups. (B-C) The NAS score and fibrosis stage of these four groups were calculated. (D) Hepatic mRNA levels for Tnf-α were measured by RT-qPCR to evaluated inflammation, GAPDH was used as control. Data was presented in mean ± SEM, ****P* < 0.001, *****P* < 0.0001; ns, not significant.

**Figure S2. Hepatocyte-specific knockout of Per2 might alleviates NASH by inhibiting ferroptosis without influencing the iron concentration.**

1. Hepatic mRNA levels for Per2 were measured by RT-qPCR in Per2^△hep^ and Per2^fl/fl^ mice, GAPDH was used as control. (B) An IPGTT test was performed at the end of 12 weeks, and the corresponding area under the curve was calculated to assess differences between Per2^△hep^ and Per2^fl/fl^ mice. (C) Body weights of Per2^△hep^ and Per2^fl/fl^ mice measured at 0, 4, 8, 12, and 16 weeks in the present experiment, and the liver/body weight ratio was measured. (D-E) The NAS score and fibrosis stage of these two groups were calculated. (F-G) Hepatic mRNA levels for Tnf-α, Il-6, Il-1β, Acsl4, Gpx4, Aifm2, and Ptgs2 were measured by RT-qPCR, GAPDH was used as control. (H) Hepatic mRNA levels for Tfr1 was measured by RT-qPCR, GAPDH was used as control. Data was presented in mean±SEM, **P* < 0.05, ***P* < 0.01; ns, not significant.

**Figure S3. Ferroptosis occurs and participates in HFHFD-induced NASH.**

(A) Hepatic mRNA levels for Acsl4, Gpx4, Aifm2, and Ptgs2 were measured by RT-qPCR, GAPDH was used as control. (B) Food intake was measured in FA+Veh and FA+Lip-1 groups. (C) Body weights of FA+Veh and FA+Lip-1 groups measured at 0, 4, 8, 12, and 16 weeks in the present experiment, and the liver/body weight ratio was measured. (D-E) The NAS score and fibrosis stage of these two groups were calculated. (F) An IPGTT test was performed the day before the mice were sacrificed, and the corresponding area under the curve was calculated to assess differences between FA+Veh and FA+Lip-1 groups. Data was presented in mean ± SEM, ***P* < 0.01, ****P* < 0.001, *****P* < 0.0001; ns, not significant.

Figure S1:


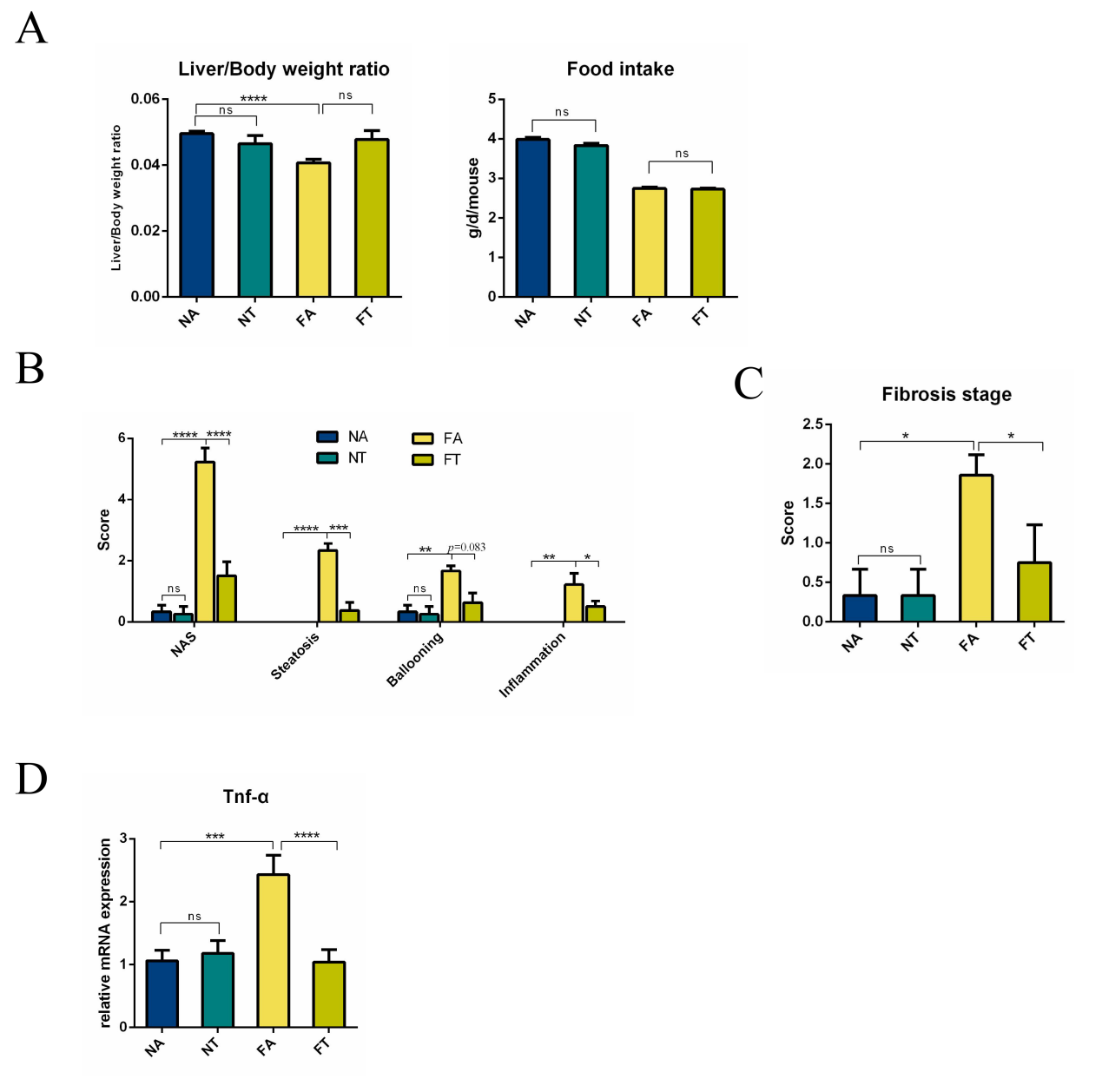


Figure S2:


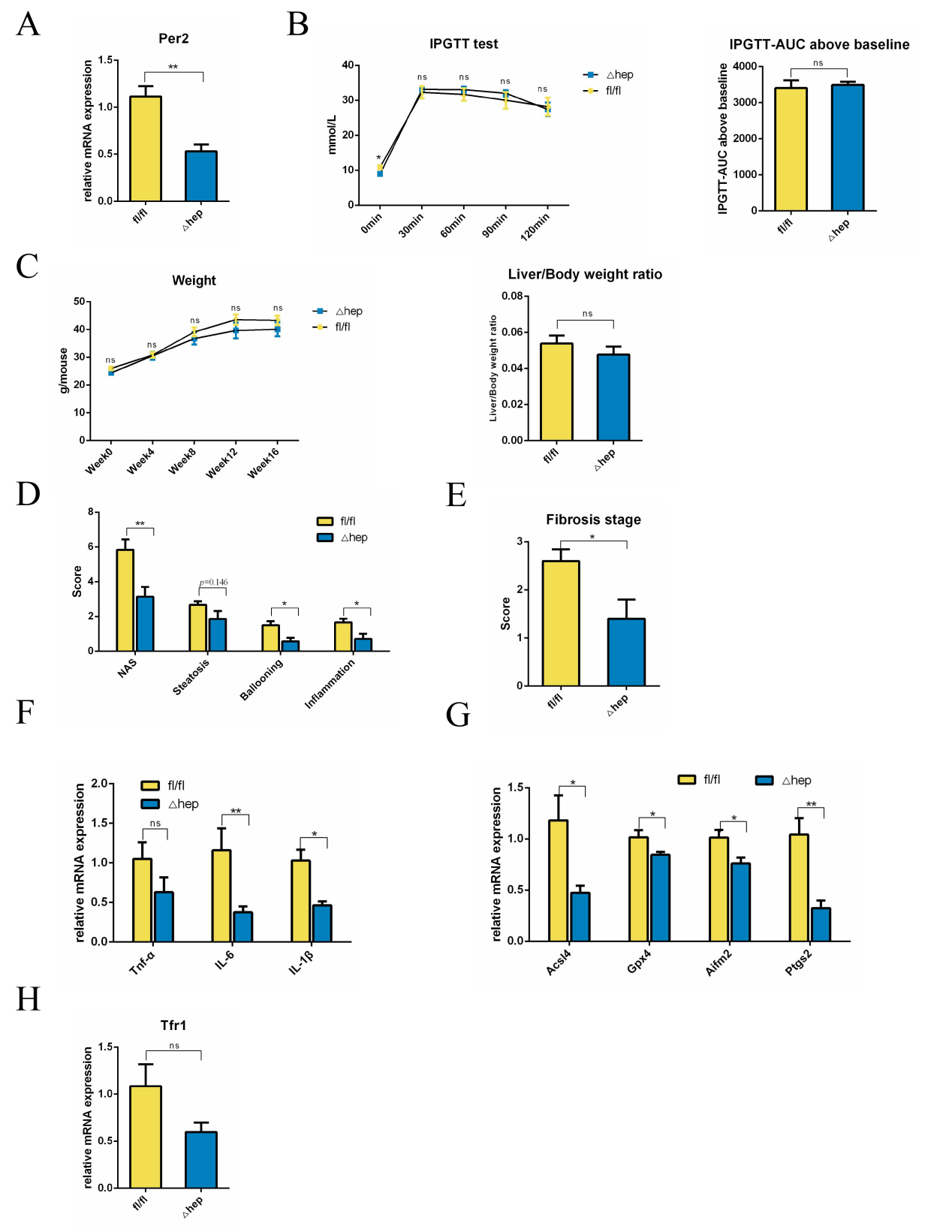


Figure S3:


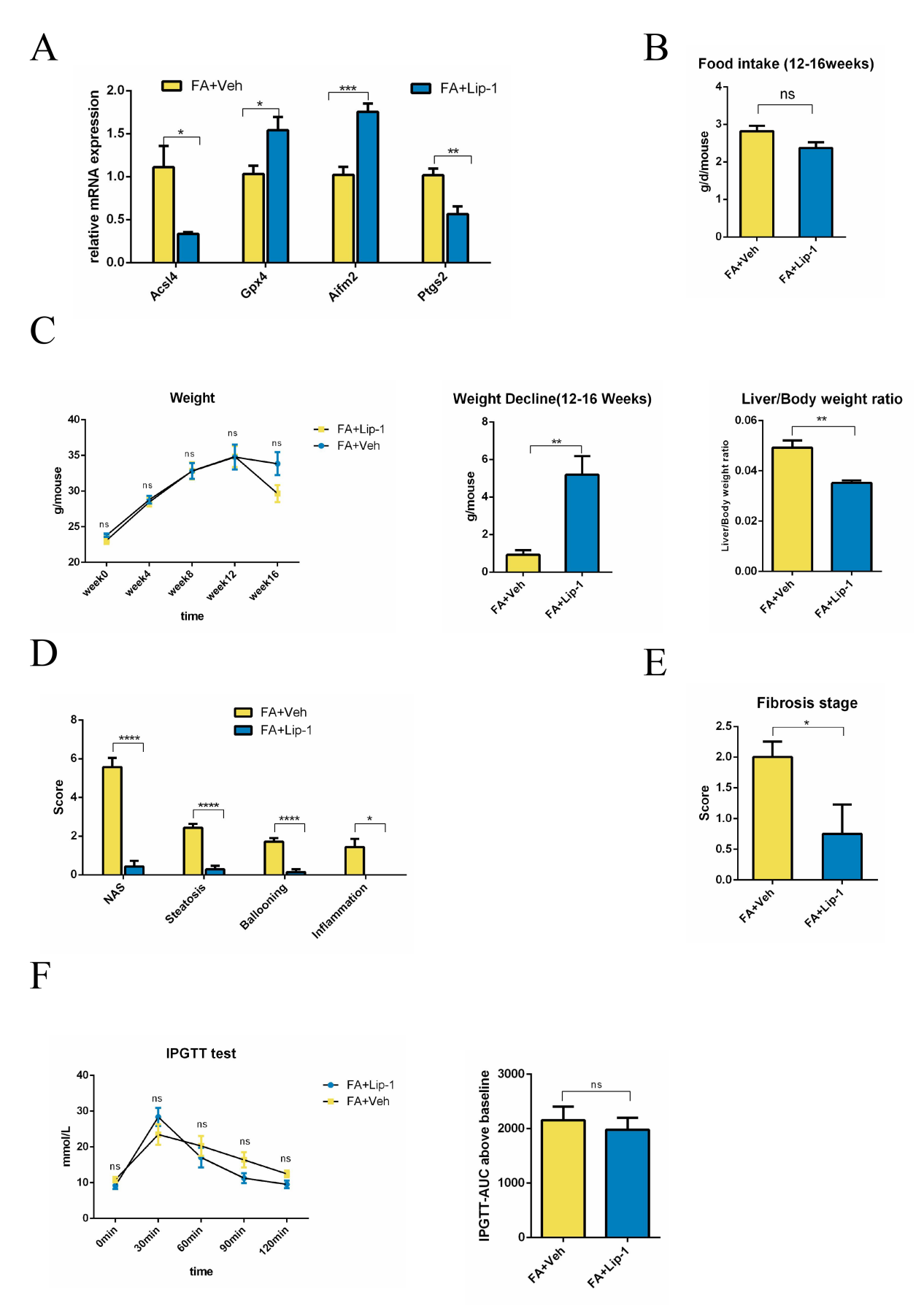

Supplement: Supplementary Materials — Some detected indices of mice in FA, FT, NA, and NT groups, Per2△hep and Per2fl/fl mice, and FA+Veh and FA+Lip-1 groups. Figure S1: TRF alleviates HFHFD-induced NASH. Figure S2: hepatocyte-specific knockout of Per2 might alleviate NASH by inhibiting ferroptosis without influencing the iron concentration. Figure S3: ferroptosis occurs and participates in HFHFD-induced NASH. [file 8063897.f1.docx]
